# Can transcranial electrical stimulation improve learning difficulties in atypical brain development? A future possibility for cognitive training

**DOI:** 10.1016/j.dcn.2013.04.001

**Published:** 2013-04-17

**Authors:** Beatrix Krause, Roi Cohen Kadosh

**Affiliations:** Department of Experimental Psychology, University of Oxford, Oxford, UK

**Keywords:** Cognitive training, Transcranial electrical stimulation, Neuroplasticity, Learning difficulties, Dyscalculia, Dyslexia, ADHD

## Abstract

Learning difficulties in atypical brain development represent serious obstacles to an individual's future achievements and can have broad societal consequences. Cognitive training can improve learning impairments only to a certain degree. Recent evidence from normal and clinical adult populations suggests that transcranial electrical stimulation (TES), a portable, painless, inexpensive, and relatively safe neuroenhancement tool, applied in conjunction with cognitive training can enhance cognitive intervention outcomes. This includes, for instance, numerical processing, language skills and response inhibition deficits commonly associated with profound learning difficulties and attention-deficit hyperactivity disorder (ADHD). The current review introduces the functional principles, current applications and promising results, and potential pitfalls of TES. Unfortunately, research in child populations is limited at present. We suggest that TES has considerable promise as a tool for increasing neuroplasticity in atypically developing children and may be an effective adjunct to cognitive training in clinical settings if it proves safe. The efficacy and both short- and long-term effects of TES on the developing brain need to be critically assessed before it can be recommended for clinical settings.

## Highlights

• Transcranial electrical stimulation (TES) can improve cognitive training effects in adults.
• TES can enhance neuroplasticity from the molecular level to the system level.
• We discuss the usage of TES with cognitive training in atypically developing children.
• We discuss the possible cognitive and physical side effects of TES.

## 1 Introduction

Learning refers to the “the acquisition of knowledge or skills through study, experience, or being taught” (“Oxford Dictionaries Online”, 2012). For the majority of individuals, this definition might be applicable. However, for a significant proportion of the population, children and adults alike, learning does not necessarily follow from studying, experiencing or being taught. The aim of the current review is to introduce the potential of transcranial electrical stimulation (TES), a non-invasive form of brain stimulation that might be used to improve learning in those who have learning difficulties based on atypical brain development, and to evaluate the relevant evidence on TES from research in adult populations.

There is currently no generally accepted definition of learning disabilities (for a recent discussion see Scanlon, 2013). However, based on the Diagnostic and Statistical Manual of Mental Disorders (5th ed.; DSM-5), learning disabilities are defined as: (1) an academic-based disorder that originates in the central nervous system, and can manifest itself in reading, writing, and/or mathematics; (2) a discrepancy in aptitude and achievement that can be identified using psychometric methods (e.g., mathematical achievement scores below the 5th percentile, despite average IQ). Further criteria for the diagnosis of learning disabilities include stipulations that: (3) learning disabilities cannot be attributed to a disparate array of difficulties such as low motivation or self-affect, albeit the individual might exhibit some of these difficulties in addition to the learning disability; (4) when assessing learning disabilities, factors such as age, gender, cultural and language group, socioeconomic factors, and level of education should be taken into account, as these factors may influence the symptom evaluation; (5) learning disabilities do not represent an “all-or-none” phenomenon, and vary in severity; (6) learning disabilities are regarded mainly as a neurodevelopmental disorder. This might suggest that other cognitive impairments can be observed to a lesser extent in other domains (Karmiloff-Smith, 1998). Furthermore, we would like to note that learning disabilities may appear in one (e.g., reading) or more (e.g., reading and mathematics) cognitive domains. While the aptitude–achievement discrepancy is currently the most established means for the identification of learning disabilities, academic performance has now been suggested as an identifier in the DSM-5 (Scanlon, 2013).

In the current review, we discuss the potential role of TES to enhance cognitive training effects in learning disabilities such as dyslexia and developmental dyscalculia (DD), which fit the aforementioned criteria. We further extend our discussion to attention-deficit hyperactivity disorder (ADHD). The inclusion of ADHD for the purpose of the current review was twofold: (1) ADHD is associated with profound difficulties in learning and involves delayed cortical development (Shaw et al., 2012); (2) current TES studies in adults have shown the efficacy in improving cognitive functions that are assumed to be impaired in ADHD (e.g., Weiss and Lavidor, 2012), which might also have implications for those who are interested in improving learning in ADHD. While according to diagnostic criteria ADHD does not constitute a learning disability, we will here refer to learning difficulties associated with atypical brain development to include ADHD and potential other developmental problems that meet the abovementioned criteria.

The current discussion will focus on DD and dyslexia, along with ADHD, as they are the best-known childhood developmental problems associated with profound difficulties in learning and atypical brain development, and evidence in healthy adults suggests that TES has favourable effects on the cognitive functions commonly impaired in these learning difficulties.

DD refers to severe difficulties in manipulating numerical information and performing arithmetic operations, and some have suggested that the deficit cannot otherwise be explained by low intelligence or by reading or attention deficits (Butterworth et al., 2011). Dyslexia, on the other hand, denotes severe difficulties in reading and text comprehension, despite an (at least) average IQ (Shaywitz, 2003). In ADHD, several domains of executive functioning can be deficient, including working memory, divided attention and response inhibition (impulsivity) (Pasini et al., 2007).

Learning difficulties have important consequences for the individual and the society they live in. The rates of unemployment, reduced income and low socioeconomic status throughout adulthood are often high in individuals with learning disabilities (Stein et al., 2011, Parsons and Bynner, 2005). Further consequences include unemployment, loss of tax payments, drug abuse, crime, special education, and depression treatment (Gross et al., 2009). In addition, the overall social and health-related consequences can be especially detrimental, as they are likely to cover the individual's entire life span (Stein et al., 2011). For instance, it has been suggested that the lack of success and achievement in individuals with learning difficulties predicts high rates of psychiatric diagnoses (Raskind et al., 1999). These factors in turn affect the state economy, in the sense that extreme annual expenses are required to counteract the deleterious societal consequences. These costs are estimated to equal nearly £2.4 billion in the UK alone for numeracy problems (Gross et al., 2009), £1.8 billion in the UK for reading disabilities (Jones et al., 2006), and $42.5 billion for ADHD in the US (Matza et al., 2005). This demonstrated burden on both the individual and the society stresses the pressing need to design successful training and intervention methods to counteract these dramatic effects of learning difficulties at the individual and societal level.

This review focuses on the application of TES together with cognitive training as a new approach to further enhance the outcome of existing cognitive training and intervention approaches to improve learning difficulties. In the following section, we will discuss different TES protocols and their underlying mechanisms. This will be followed by an overview of the current evidence on improvements in cognitive training using TES in both healthy and clinical adult populations. Besides a critical discussion on the interpretation of results and associated pitfalls and risks of the method, we will also outline the potential benefits for TES as a future intervention technique in children with learning difficulties and discuss its potential role in ameliorating associated learning deficits.

## 2 An introduction to TES

Two thousand years ago, physicians applied the electrical current emitted by torpedo fish to alleviate a variety of medical symptoms. As known from Islamic and Greco-Roman writings, headaches, epileptic seizures, pain and other symptoms were treated by applying the electrical current of the fish to the symptom site (Finger and Piccolino, 2011). Almost two millenia later, in the 18th century, applying electricity to the head was re-pioneered as a cure for mental illnesses such as epilepsy and hysteria (Gilman, 2008). It has since been refined and has become a widely applied technique in both scientific and clinical rehabilitation settings. The most frequently used forms are deep brain stimulation (DBS) and TES. In DBS, implanted electrodes stimulate specific cortical and subcortical regions to treat a variety of neuropsychiatric conditions, such as Parkinson's disease (Huys et al., 2012). In TES, cortical brain areas are targeted from the outside surface of the scalp by using one or more electrodes (Im et al., 2012, Nitsche and Paulus, 2001). Due to its non-invasiveness and its possibility to induce long-term synaptic plasticity, TES has considerable potential as a rehabilitation method for enhancing cognitive performance in more moderate neurological and clinical conditions (Cohen Kadosh, 2013, Nitsche et al., 2008), including learning difficulties and atypical cortical development in children (Cohen Kadosh et al., 2012b).

TES has currently only few known, minor side effects, such as skin irritation and nausea (see Table 1). In healthy adults, no cases of seizures have been reported to date (Poreisz et al., 2007). It is important to stress, however, that the associated risk of TES should not automatically be inferred from adult to child samples and evidence from paediatric samples is very limited (see Mattai et al., 2011, Schneider and Hopp, 2011, for a detailed discussion, see Section 5).

**Table 1:** Potential known and possible consequences caused by TES in the developing brain. Unknown factors need to receive scientific attention and careful exploration in order to be able to label the method ‘safe’ in paediatric population.

Population	Adults		Children	
	Short-term effects	Long-term effects	Potential short-term effects	Potential long-term effects
Physical tolerability	Tingling (70.6%), itching (30.4%), burning sensation (21.6%), pain (15.7%), skin irritation (redness), headaches (4.9%), fatigue (35.3%) (Poreisz et al., 2007)	None reported	Induction of seizures	Neurological impairments and/or risk for epilepsy
Cognitive effects associated with stimulated brain region	Task-specific improvements or reductions in performance (Table 2, Table 3)	Persistence of improvements on experimental task (up to 6 months: Cohen Kadosh et al., 2010)	Maladaptation and dysfunctional integration of neural network under development	Irreversible shaping of the network leading to faulty cognitive functioning, or transfer effects to another cognitive domain
Cognitive effects associated with other brain regions	Unknown	Unknown	Remote effects or secondary plastic changes (e.g., by lateral inhibition of the stimulated region) (Zheng et al., 2011)	Unintended cognitive impairments compromised by dominant stimulated brain region

The TES current, which is typically delivered at 1–2 mA (Table 2, Table 3), is applied by a battery-driven current generator (e.g., a 9 V battery), through electrodes that are fixed to the scalp surface by straps or a cap. The electrodes are covered by rubber sponges and are usually soaked in saline solution to enhance conductivity with the skin (Zaghi et al., 2010). The exact locations of the target stimulation regions are usually determined using the international 10–20 system for EEG electrode recording (Auvichayapat and Auvichayapat, 2011). One or more electrodes are placed over the to-be-stimulated site, with a reference electrode elsewhere on the head or body, and current flows from one to the other. In the majority of experiments, behavioural performance during the stimulation is contrasted against the performance during the corresponding sham (placebo) stimulation (Table 2, Table 3). In some cases, a control brain region is used (e.g., Bolognini et al., 2010, Sparing et al., 2008). The participant, and in some cases the experimenter, is blind to the respective condition. One of the major advantages of TES is that its sham condition is indistinguishable from the real stimulation to the person receiving it (Gandiga et al., 2006), as pre-programmed settings allow an initial stimulation period (e.g., 30 s), which then ramps down and offsets the current. The participant experiences the skin sensations typical of TES during this initial period and thereby remains unaware of the real condition.

**Table 2:** TES studies on cognitive functions involving clinical populations. Rt: right; lt.: left; RT: reaction time; ACC: accuracy; ATDCS: anodal transcranial direct current stimulation; CTDCS: cathodal transcranial direct current stimulation; (hf-/lf-) TRNS: (high-frequency/low-frequency) transcranial random noise stimulation; RALC: rt.-anodal, lt.-cathodal; RCLA: rt.-cathodal, lt.-anodal; WM: working memory; SMA: supplemental motor area; M1: primary motor cortex; STG: superior temporal gyrus; IFG: inferior frontal gyrus; DLPFC: dorsolateral prefrontal cortex; PC: parietal cortex; PPC: posterior parietal cortex; IPL: inferior parietal lobe; SPL: superior parietal lobe; WS: within-subject design; BS: between-subject design; N/A: detailed information not available. Effect sizes have been estimated whenever not provided in the original paper. Cohen's *d*: 0.2 is considered as a ‘*small*’ *effect size, d* ≤ 0.5 represents a ‘*moderate*’ *effect size* and *d* ≤ 0.8 is a ‘*large*’ *effect size.*

Authors	Population	*N*	Mean age (in years)	Sex	Cognitive function	TES	Amp	Electrode size	mA/cm^2^
*Language*									
You et al. (2011)	Sub-acute stroke patients with global aphasia	21	67, 48–82	9f, 12m	Speech	ATDCS, CTDCS, sham	2 mA	7 cm × 5 cm	0.06
Marangolo et al. (2011)	Stroke patients with aphasia	3	N/A	1f, 2m	Speech	ATDCS, sham	1 mA	7 cm × 5 cm	0.03
Fiori et al. (2011)	Healthy subjects, stroke patients with aphasia	10 healthy, 3 stroke	Healthy: 55 ± 7.9, 45–70	Healthy: 3f, 7m; patients: 3m	Word retrieval	ATDCS sham (WS)	1 mA	7 cm × 5 cm	0.03
Fridriksson et al. (2011)	Chronic stroke patients with aphasia	8	68.13 ± 10.40, 53–79	N/A	Naming	ATDCS, sham (WS)	1 mA	N/A	N/A
Vines et al. (2011)	Lt. frontal stroke patients with aphasia	6	56.2, 30–81	6 m	Speech fluency	ATDCS, sham (WS)	1.2 mA	16.3 cm^2^, reference electrode 30 cm^2^	0.07
Schneider and Hopp (2011)	Minimally verbal children with autism	10	9.8 ± 4.4, 6–21	2f, 8 m	Syntax acquisition	TDCS	2 mA	5 cm × 5 cm	0.08

Authors	Stimulation sites	Stimulation duration	Double blind	No dropouts	Training	Results	Effect size (Cohen's *d*)
*Language*							
You et al. (2011)	ATDCS: lt. STG (Wernicke's area, CP5), CTDCS: rt. STG (CP6), sham group: CP5	30 min	+	−	5 times a week for 2 weeks	• ATDCS improved: • aphasia quotients • spontaneous speech • CTDCS improved: • auditory verbal comprehension	CTDCS vs. ATDCS *d* = 1.04 CTDCS vs. sham *d* = 1.07
Marangolo et al. (2011)	Lt. IFG (Broca's area)	20 min	+	+	5 consec. days: repetition task	• ATDCS improved speech ACC	N/A
Fiori et al. (2011)	Healthy subjects: TDCS/sham over Wernicke's area (CP5), or TDCS to rt. occipito-parietal area (O2); patients: 5 consec. days ATDCS/sham	20 min	+	+ (retest −)	3 days of training with 6 days in between each/ aphasic patients: 5 consec. days for both ATDCS and sham	• ATDCS improved naming ACC and RTs	Healthy: anodal < sham: *d* = .91; right anodal = sham: *d* = .37; left anodal < right anodal: *d* = 1.18 Patients: anodal < sham: *d* = .43; day 5 < day 1 anodal: *d* = .52 (sham: *d* = .06); day 5 anodal < day 5 sham: *d* = .57
Fridriksson et al. (2011)	Lt. posterior cortex, reference cathode on rt. forehead	20 min	+	N/A	10 sessions of anomia training (5 consec. days per stimulation condition)	• ATDCS improved RT in naming task for trained items (stable at 3 weeks follow-up • 75% of patients had stable ACC and RT at 1 and 3 weeks follow-up	
Vines et al. (2011)	Rt. posterior IFG (2.5 cm posterior to F8)	20 min	+	+	3 consec. days of training, 1 week apart	• ATDCS improved speech fluency	Percentage change anodal > sham: *d* = 1.98
Schneider and Hopp (2011)	Lt. DLPFC (F3), cathode rt. supraorbital region	30 min	− (only pre vs. post)	+	Syntax and vocabulary testing	• TDCS improved syntax acquisition from pre- to posttest	Mean vocabulary: post-TDCS > pre-TDCS: *d* = .96; mean syntax: post-TDCS > pre-TDCS: *d* = 2.78

Authors	Population	*N*	Mean age (in years)	Sex	Cognitive function	TES	Amp	Electrode size	mA/cm^2^
*Memory*									
Ferrucci et al. (2008)	Alzheimer patients	10	75.2 ± 7.3	7f, 3m	Word recognition memory and visual attention	ATDCS, CTDCS, sham (WS)	1.5 mA	N/A	N/A
Boggio et al. (2012)	Alzheimer patients	15	79.05 ± 8.2	7f, 8m	Visual recognition memory	TDCS, sham (WS)	2 mA	35 cm^2^, deltoid 64 cm^2^	0.06

Authors	Stimulation sites	Stimulation duration	Double blind	No dropouts	Training	Results	Effect size (Cohen's *d*)
*Memory*							
Ferrucci et al. (2008)	Bilateral temporo-parietal areas (P3-T5 and P6-T4)	15 min	+	+	3 days of training per condition (10 days apart); conditions 71 days apart on average	• ATDCS: recognition memory ACC improved • CTDCS: recognition memory ACC reduced • Sham: recognition memory ACC unchanged	Post-ATDCS > pre-ATDCS: *d* = .89; post-CTDCS < pre-CTDCS: *d* = 1.07; sham unchanged (*d* = .11)
Boggio et al. (2012)	Temporal lobe (T3, T4), reference rt. deltoid muscle	30 min	+	+	5 days of training per condition	• ATDCS improved visual recognition performance • Sham: visual recognition reduced • Persistent at 4 weeks follow-up	Change from baseline TDCS: end of testing: *d* = .35; 1 week later: *d* = 0; 4 weeks later: *d* = .28; sham: end of testing: *d* = .23; 1 week later: *d* = .02; 4 weeks later: *d* = .05

**Table 3:** TES studies on cognitive functions involving normal populations. Rt.: right; lt.: left; RT: reaction time; ACC: accuracy; ATDCS: anodal transcranial direct current stimulation; CTDCS: cathodal transcranial direct current stimulation; (hf-/lf) TRNS: (high-frequency/low-frequency) transcranial random noise stimulation; RALC: rt.-anodal, lt.-cathodal; RCLA: rt.-cathodal, lt.-anodal; WM: working memory; SMA: supplemental motor area; M1: primary motor cortex; STG: superior temporal gyrus; IFG: inferior frontal gyrus; DLPFC: dorsolateral prefrontal cortex; PC: parietal cortex; PPC: posterior parietal cortex; IPL: inferior parietal lobe; SPL: superior parietal lobe; WS: within-subject design; BS: between-subject design; N/A: detailed information not available. Effect sizes have been estimated whenever it has not been provided in the original paper. Cohen's *d* = 0.2 is considered as a ‘*small*’ *effect size, d* ≤ 0.5 represents a ‘*moderate*’ *effect size* and *d* ≤ 0.8 is a ‘*large*’ *effect size.*

Authors	*N*	Mean age (in years)	Sex	Cognitive function	TES	Amp	Electrode size
*Numerical abilities*							
Cohen Kadosh et al. (2010)	15	Range 20–22	N/A	Numerical abilities	RALC, RCLA, sham	1 mA	3 cm × 3 cm
Iuculano and Cohen Kadosh (2013)	19	Range 20–31	9f, 10m	Numerical abilities	RALC (DLPFC), RCLA (PPC), sham	1 mA	3 cm^2^

Authors	mA/cm^2^	Double blind	No dropouts	Stimulation sites	Stimulation duration	Training	Results	Effect size (Cohen's *d*)
*Numerical abilities*								
Cohen Kadosh et al. (2010)	0.1	−	+ (dropout only at 6 month follow-up)	Lt. and rt. PC (P3, P4)	20 min	6 consec. days	• RALC increased automaticity on numerical Stroop • RCLA decreased performance • Sham in between RALC and RCLA • Stable at 6 month follow-up	*d* = 1.09
Iuculano and Cohen Kadosh (2013)	0.1	N/A	+	PPC (P3, P4), DLPFC (F3, F4)	20 min	Single session (120 min)	• RCLA to PPC improved learning rates for articificial numbers compared to sham, RALC to DLPFC decreased learning rate • RALC to DLPFC improved Stroop automaticity compared to sham, RCLA to PPC decreased Stroop automaticity	Learning rate: *d* = .85; Stroop automaticity: *d* = .55

Authors	*N*	Mean age (in years)	Sex	Cognitive function	TES	Amp	Electrode size
Vision							
Fertonani et al. (2011)	84	21.7 ± 2.5, 19–30	42f, 42m	Orientation discrimination	hf-TRNS, lf-TRNS; ATDCS CTDCS, sham, Cz	1.5 mA	16 cm^2^
Bolognini et al. (2010)	20	24, range 20–26	16f, 4m	Multi-sensory visual field exploration	ATDCS, sham (WS)	2 mA	7 cm × 5 cm

Authors	mA/cm^2^	Double blind	No dropouts	Stimulation sites	Stimulation duration	Training	Results	Effect size (Cohen's *d*)
Vision								
Fertonani et al. (2011)	0.09	N/A (not for Hf-TRNS condition)	+	Primary visual cortex (V1, 3.5 cm above the inion), Cz for hf-TRNS	22 min	N/A	• TRNS enhanced learning rate compared to ATDCS	ATDCS < hf-TRNS: *d* = .7
Bolognini et al. (2010)	0.06	+	+	Lt. PPS (P3), rt. PPS (P4)	30 min	1 session per condition, 1 week apart	• Rt. ATDCS improved visual exploration	*d* = .28

Authors	*N*	Mean age (in years)	Sex	Cognitive function	TES	Amp	Electrode size
Memory							
Tecchio et al. (2010)	44	29 ± 5	22f, 25m	Procedural consolidation	ATDCS, sham	1 mA	7 cm × 5 cm
Gladwin et al. (2012)	14	22 ± 3		Selective attention in WM	ATDCS, sham (WS)	1 mA	7 cm × 5 cm
Teo et al. (2011)	12	27.23, 22–55 ± 9.18	7f, 5m	WM	ATDCS (1 mA), ATDCS (2 mA), sham	1 mA, 2 mA	35 cm^2^
Sandrini et al. (2012)	27	25 ± 2, 20–30	4f, 5m (per group)	WM	Lt.-anodal-rt.-cathodal, sham (BS)	1.5 mA	7 cm × 5 cm
Berryhill et al. (2010)	11	25	5f, 6m	WM	ATDCS, CTDCS, sham (WS)	1.5 mA	7 cm × 5 cm
Mulquiney et al. (2011)	10	29.4 ± 5.8	6f, 4m	WM	ATDCS, hf-TRNS, sham (WS)	1 mA	7 cm × 5 cm
Ohn et al. (2008)	15	26.5 ± 3.5	10f, 5m	WM	ATDCS, sham (WS)	1 mA	5 cm × 5 cm

Authors	mA/cm^2^	Double blind	No dropouts	Stimulation sites	Stimulation duration	Training	Results	Effect size (Cohen's *d*)
Memory								
Tecchio et al. (2010)	0.03	N/A	−	Rt. M1 (C4)	15 min	N/A	• ATDCS enhanced early consolidation of trained finger tapping sequences	N/A
Gladwin et al. (2012)	0.03	+ (blinding compromised)	+	Anode lt. DLPFC, cathode rt. orbit	10 min	1 session per condition, 30 minutapart (50 min from offset to onset)	• ATDCS improved RT in the presence of incorrect distracters	3 items: TDCS(RT) < sham(RT): *d* = .27; 5 items: TDCS_RT_ < sham(RT) *d* = .33; 7 items: TDCS(RT) < sham(RT): *d* = .53
Teo et al. (2011)	0.03, 0.06	+	−	Lt. DLPFC (F3)	20 min	1 session per condition, 1 week apart	• No improvements in ACC but interaction between current strength and RT	2 mA_RT_ < sham(RT): *d* = .31
Sandrini et al. (2012)	0.04	N/A	+	PPC (P3 and P4)	13 min	Single-session	• Interaction stim. condition and task: LARC-TDCS abolished reductions in RT measured in 1-back task; LCRA-TDCS abolished reductions in RT on 2-back task, compared to RCLA-TDCS and sham	1-back task: LHA-RHC versus sham: *d* = .72; LHC-RHA vs. LHA-RHC: *d* = .99; sham vs. LHC-RHA: *d* = .27; 2-back task: LHC-RHA vs. sham: *d* = .1.79; LHA-RHC vs. LHC-RHA: *d* = .72; sham vs. LHA-RHC: *d* = .08
Berryhill et al. (2010)	0.04	N/A	−	Active electrode rt. inferior PC (P4), cathode lt. cheek	10 min	1 session per condition on separate days	• CTDCS reduced WM recognition performance compared to sham	CTDCS < sham: *d* = .39 (rough estimation)
Mulquiney et al. (2011)	0.03	−	−	Lt. DLPFC (F3)	10 min	3 × 1 session, 1 week apart	• ATDCS decreased RT in the 2-back task	TDCS pre- vs. post: *d* = .36; TDCS vs. sham: *d* = .45
Ohn et al. (2008)	0.04	−	+	Lt. DlPFC (F3), cathode over contralateral rt. supraorbital area	20 min	3-back WM task	• ATDCS enhanced ACC, effect even larger after 30 min; maintained for at least another 30 min	ATDCS vs. sham: baseline: *d* = .22; after 10 min: *d* = .15; after 20 min; *d* = .58; after 30 min: *d* = .84; 30 min after completion: *d* = .64

Authors	*N*	Mean age (in years)	Sex	Cognitive function	TES	Amp	Electrode size
Attention							
Hsu et al. (2011)	28	Pre-SMA group: 22.1, range 20–26 M1: 21.79, 18–27	6f, 8m; 6f, 8m (control)	Inhibitory control (addressing ADHD)	ATDCS, CTDCS	1.5 mA	4 cm × 4 cm
Jacobson et al. (2012a)	12 + 12 controls	26.7 ± 8.7, *control*: 24.2 ± 0.9	7f, 5m, *control:* 7f, 5m	Recognition memory	ATDCS, CTDCS (WS)	1 mA	5 cm × 5 cm
Ditye et al. (2012)	22	ATDCS 23.58 ± 4.16	ATDCS 7f, 3m; no stimulation 7f, 5m	Behavioural inhibition	ATDCS, no stimulation (BS)	1.5 mA	7 cm × 5 cm
Weiss and Lavidor (2012)	30	26.5 ± 5.9, 18–48	20f, 10m	Attention	ATDCS, CTDCS, sham (BS)	1.5 mA	Active 4 cm × 4 cm, passive 7 cm × 5 cm
Dockery et al. (2009)	24	24 ± 3.16, 19–32	19f, 5m	Executive planning	ATDCS, CTDCS, sham (WS)	1 mA	35 cm^2^
Bolognini et al. (2010)	48	*Exp. 1*: 24 ± 6; *exp. 2*: 22 ± 5; *exp.3*: 25 ± 4	*Exp. 1*; 10f, 6m; *exp. 2*: 9f, 7m; *exp.3*: 15f, 1m	Audio- and visual spatial orienting	*Exp. 1*: ATDCS, *exp. 2*: sham; *exp. 3*: ATDCS to control region	2 mA	35 cm^2^

Authors	mA/cm^2^	Double blind	No dropouts	Stimulation sites	Stimulation duration	Training	Results	Effect size (Cohen's *d*)
Attention								
Hsu et al. (2011)	0.09	−	−	Pre-SMA, superior middle prefrontal (exp.) vs. M1 (control, Fz)	10 mA	N/A	• ATDCS improved inhibitory control • CTDCS showed a tendency towards reducing inhibitory control	ATDCS vs. CTDCS: *d* = 3.25; ATDCS vs. no TDCS: *d* = 1.77
Jacobson et al. (2012a)	0.04	+	+	Rt.-IPL-cathode- lt.-IPS/SPL-anode (P3 + P6)+, rt.-IPL-anode + lt.-IPS/SPL-cathode (P6)	10 min	N/A	• Lt. anodal IPS/SPL-rt. cathodal IPL enhanced recognition memory	*d* = .54
Ditye et al. (2012)	0.04	−	+	Rt. IFG (anode between T4-Fz and F8-Cz), cathode lt. orbitofrontal cortex (above lt. eyebrow)	15 min	8 min of SST training on 5 consec. days	• TDCS improved response inhibition	Day 3: ATDCS < control: *d* = .29; day 4 ATDCS < control: *d* = 3.24 (estimated)
Weiss and Lavidor (2012)	0.09	N/A	−	Rt. PPC (P4), lt. supraorbital forehead	15 min	Single session	• CTDCS improved flanker processing compared to A-TDCS and sham	CTDCS(Flanker effect) > ATDCS(Flanker effect): *d* = .27; CTDCS(Flanker effect) > sham(Flanker effect): *d* = .43
Dockery et al. (2009)	0.03	−	−	Lt. DLPFC (F3), rt. orbit	15 min	1 session per stimulation condition, 1 week apart	• CTDCS improved planning during acquisition and consolidation if preceded by ATDCS • ATDCS improved planning in later sessions if preceded by • CTDCS persistent performance at 6 and 12 months follow-up (retested under sham)	RT: CTDCS: session (S) 1: *d* = .46; S2: *d* = .38; S3: *d* = −.23; ATDCS: S1: *d* = −.22; S2:.12; S3: *d* = .67; ACC: ATDCS: S1: *d* = −.12; S2: *d* = .38; S3: *d* = .48; CTDCS: S1: *d* = .84; S2: *d* = .22; S3: *d* = −.45
Bolognini et al. (2010)	0.06	+	−	A-TDCS to rt. PPC (P4); sham rt. PPC (P4); A-TDCS to rt. V1 (O2); reference to contralateral deltoid muscle	15 min	2 sessions per condition	• A-TDCS to rt. PPC improved orienting to both modality-specific and crossmodal task stimuli, particularly the probabilistic audiovisual redundant signal effect (RSE)	N/A

Authors	*N*	Mean age (in years)	Sex	Cognitive function	TES	Amp	Electrode size
Language							
Turkeltaub et al. (2011)	23	26.7, 20–50	15f, 10m	Reading efficiency	ATDCS, sham	1.5 mA	5 cm × 5 cm
Flöel et al. (2008)	19	25.6 ± 2.7, 22–32	9f, 10m	Associative language learning	ATDCS, CTDCS, sham (WS)	1 mA	7 cm × 5 cm
Holland et al. (2011)	10	69, range 62–74	7f, 3m	Speech; naming	ATDCS, sham (WS)	2 mA	7 cm × 5 cm
Cattaneo et al. (2011)	10	23.6 ± 3.2	N/A	Semantic and phonemic fluency	ATDCS, sham (WS)	2 mA	7 cm × 5 cm
Sparing et al. (2008)	15	26.9 ± 3.7	5f, 10m	Picture naming	ATDCS to CP5, CTDCS to CP5; *control*: ATDCS to CP6, sham to CP5	2 mA	7 cm × 5 cm
De Vries et al. (2010)	44 + 10 controls	22.6 ± 2.1; *control*: 23.7 ± 2.4	19f, 25m; *control*: 5f, 5m	Syntactic violation detection and rule-based knowledge	ATDCS, sham, *control:* ATDCS over Cz (BS)	1 mA	7 cm × 5 cm, ref. 10 cm × 10 cm

Authors	mA/cm^2^	Double blind	No dropouts	Stimulation sites	Stimulation duration	Training	Results	Effect size (Cohen's *d*)
Language								
Turkeltaub et al. (2011)	0.06	−	+	Lt. posterior temoral cortex (pTC) (in between T7 and TP7); cathode rt. pTC between T8 and TP8	20 min	2 sessions each	• Word reading efficiency was improved in below-average readers	*d* = .49
Flöel et al. (2008)	0.03	+	+	Lt. posterior peri-sylvian area (Wernicke's area, Cp5), reference on contralateral supraorbital region	20 min	1 session per stim. condition, 7 days apart	• ATDCS increased ACC and associative learning speed	ATDCS > sham: *d* = 1.35; CTDCS < sham: *d* = .26
Holland et al. (2011)	0.06	N/A	+	Lt. IFG (Broca's area, FC5)	20 min	Single-session	• ATDCS increased naming speed • ATDCS decreased BOLD signal in Broca's area	ATDCS (RT) < sham (RT): *d* = .27
Cattaneo et al. (2011)	0.06	−	+	Anode Broca's area (crossing point between T3-Fz and F7-Cz)	20 min	1 session per condition	• ATDCS improved word production in both semantic and phonemic tasks compared to sham	TDCS > sham: *d* = 1.16
Sparing et al. (2008)	0.06	N/A	+	Wernicke's area (CP5), rt. posterior perisylvian region (PPR) (CP6), reference Cz	7 min	1 session per condition, at least 4 h apart	• ATDCS over Wernicke's area enhanced picture-naming latency	ATDCS(RT) vs. sham(RT): *d* = .44; ATDCS(RT) vs. sham(RT): *d* = .47; picture naming latency CP6 > CP5: *d* = .34
De Vries et al. (2010)	0.03	N/A	−	Broca's area, ref. rt. Supraorbital region	20 min	Single session	• ATDCS to Broca's area improved the detection of syntactic violations	TDCS > sham: *d* = 1.57

It is important to make a clear distinction between TES and a similar, but more familiar, neuroscientific technique, called transcranial magnetic stimulation (TMS). TMS and TES both have advantages and disadvantages, depending on the intended research field and parameters of use (for a review see Wagner et al., 2007). In TMS, magnetic pulses are administered to the scalp surface with a magnetic coil, which induces a magnetic field in the cortical region below (Walsh and Pascual-Leone, 2003). This magnetic field leads directly to the depolarization of cortical neurons and thus leads to action potentials, which can be followed by an observable, short-term change in the behavioural response (e.g., finger movement after M1 stimulation) (Schläpfer and Kayser, 2012). This makes TMS particularly useful to explore the causal role of a given cortical region in cognition and functioning. Depending on the preferred parameters, it can induce either immediate activation by triggering action potentials in the stimulated region, or temporary virtual ‘lesions’ by inhibiting activation in the given region (Ruff et al., 2009). The direct change in the behavioural response reveals whether the targeted region is involved in the processing of the task at hand.

In contrast to TMS, TES affects the stimulated neurons in a more subtle fashion. Namely, it modulates neuron membrane potentials—and thus concurrent cortical excitability—during task execution, and thereby possibly induces more long-lasting cognitive changes (Zaghi et al., 2010). These long-term changes might therefore have a higher rehabilitative value than TMS for individuals with cognitive dysfunctions (Brunoni et al., 2012). In addition, the repeated administration of TMS increases the chance for epileptic seizures (Wassermann, 1998). Therefore, TES seems to be more favourable in cases where repeated use is required, such as in multiple cognitive training sessions.

Another advantage of TES is that it is more comfortable for the receiving individual than TMS. TMS generates loud clicking noises that are associated with “tapping” sensations on the skin, with a high potential for eliciting facial twitches in some areas (Wagner et al., 2007). Moreover, the discomfort induced by unwanted action potentials in the muscles under the skin at the stimulation position can also affect the performance on cognitive tasks (Abler et al., 2005).

**Fig. 1:**
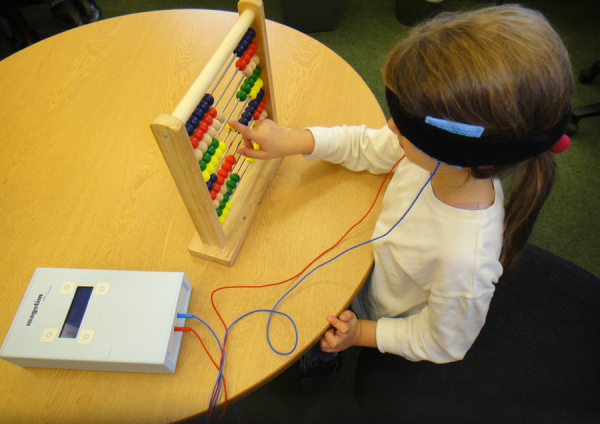
Electrode placement for the lateral prefrontal cortex stimulation. The electrodes are wired with the stimulator (bottom left), which is a 9 V battery pack and are held in place using a headband.

Reprinted from Current Biology, 22, Cohen Kadosh, R., Levy, N., O'Shea, J., Shea, N., & Savulescu, J. The neuroethics of non-invasive brain stimulation, R108-R111 (2012), with permission from Elsevier.

However, one of the potential advantages of TMS over TES is its higher spatial and temporal resolution (Wagner et al., 2007). Nevertheless, there is no evidence yet that these advantages are critical for inducing neuroplasticity during learning. Since TES training is repeatedly administered over an extended period of time, a temporal resolution of 1 millisecond (vs. 5 min) may not add any benefit in this case. The relatively poor focality of TES ranges in the order of centimetres, but at the same time diminishes the necessity for complex and expensive functional MRI- or MRI-based neuronavigation systems, as is used in TMS (Sack et al., 2009). Compared to the costly technical equipment, as well as the required advanced knowledge and skills in its administration of TMS, TES is relatively inexpensive (simple devices can start at around £500). Furthermore, unlike TMS, TES equipment is compact and portable and does not require extensive training to administer (Brunoni et al., 2012, Cohen Kadosh et al., 2012b). Overall, these factors make TES more suitable for experimental and clinical settings than TMS, especially for the purpose of repeated cognitive training and for modulating neuroplasticity (Fig. 1).

### 2.1 TES: types and mechanisms

Currently, there are three main types of TES: (1) transcranial direct current stimulation (TDCS); (2) transcranial random noise stimulation (TRNS); and (3) transcranial alternating current stimulation (TACS). TDCS is the most widely used to date and offers two subtypes of stimulation: (1) *anodal stimulation* (ATDCS) enhances neuronal firing by inducing depolarizations (neuronal excitation); and (2) *cathodal stimulation* (CTDCS) depresses the firing rate and hyperpolarizes neuronal firing (inhibition) (Nitsche and Paulus, 2000). In other words, in ATDCS, the firing threshold of neurons is decreased, such that the neurons in the stimulated area require less input to fire. In CTDCS, the firing threshold of neurons is increased, such that the stimulated area becomes inhibited and requires more input. Depending on the intended goal (facilitation or inhibition), ATDCS or CTDCS can be selected to either enhance or reduce neuronal excitability and thereby modulate cognitive abilities.

TES studies with healthy adults and patients with brain damage and/or cognitive dysfunctions demonstrate how neural circuits can be modulated during cognitive training to improve deficient cognitive functioning; in some instances, long-lasting effects have even been demonstrated (Table 2, Table 3). The modulatory effect of ATDCS reduces regional levels of the inhibitory neurotransmitter *gamma*-Aminobutyric acid (GABA), whereas CTDCS decreases glutamate transmission in the motor cortex in conjunction with a simple motor task (Stagg et al., 2009). GABA levels correlate negatively with learning (Floyer-Lea et al., 2006) and are therefore likely to play a role in learning and cognitive enhancement associated with TDCS. Furthermore, animal studies have shown that ATDCS can enhance the secretion of brain-derived neurotrophic factor (BDNF), which is a crucial growth factor in synaptic learning. This in turn can modulate long-term potentiation (LTP) (Castillo et al., 2011, Fritsch et al., 2010), which is known to be mediated by NMDA-receptor activity (Nitsche et al., 2003). Whereas most cognitive functions (e.g., reading, speech, decision making, or arithmetic) are aimed to be improved using ATDCS by enhancing cortical excitability (Cohen Kadosh et al., 2010, Flöel et al., 2008, Hecht et al., 2010, Holland et al., 2011), certain frontal functions, such as response inhibition, can be improved by suppressing the neural activity in related networks using CTDCS (see e.g., Hsu et al., 2011). In this case, the inhibition functions as a filter on impulsive responses.

TRNS is a recently introduced version of TES, which applies the current at quickly varying frequency bands (Terney et al., 2008). In this method, the stimulation emanates from both electrodes simultaneously (which are the same electrodes used for TDCS) and can excite both stimulated brain regions at the same time. There are two advantages for TRNS over TDCS: (1) due to its lower probability of causing skin sensations compared to TDCS, TRNS can provide even superior blinding conditions (Ambrus et al., 2010); (2) it can modulate two brain regions simultaneously without facing inhibitory cathodal effects under the second electrode. For example, TRNS would be desirable in the case of arithmetic training where the bilateral dorsolateral prefrontal cortex (DLPFC) is heavily involved (Zamarian et al., 2009). Indeed, it has been found that the application of TRNS to the bilateral DLPFC during 5 days of arithmetic training increased the learning rate compared to sham stimulation (Snowball et al., 2013). In both TRNS and TDCS, even brief stimulation durations (e.g., 10 min) can lead to excitability increases both during and up to 60 min after stimulation, and have been demonstrated to facilitate the effects of learning, compared to sham stimulation (Terney et al., 2008).

Fertonani and associates (Fertonani et al., 2011) suggested that the effects induced by TRNS can be explained by a phenomenon called stochastic resonance. According to the stochastic resonance framework, the presence of neuronal noise can make neurons more sensitive to a given range of weak inputs.

Since the brain is a nonlinear system, it can use the noise to enhance performance (Moss et al., 2004). Another explanation, which has been offered by Terney et al. (2008), is that by using TRNS, sodium channel activity can be augmented. According to this explanation, sodium channels would reopen (repolarisation) within a shorter time frame after depolarisation under TRNS compared to non-TRNS and thereby make the neuron ready for repeated excitation. However, as TRNS is a recent version of TES, such suggestions as to the operating mechanism are currently only hypotheses and supporting experimental evidence is required.

The third type of TES is TACS. In contrast to the quickly varying random frequencies in TRNS, TACS stimulates at a fixed frequency (e.g., 10 Hz). The stimulation is applied at low intensities and the direction of the current is constantly alternating, so that each electrode interchangeably serves as either anode or cathode (Zaghi et al., 2010). The ideal stimulation frequency for behavioural effects hereby depends on the current cortical oscillation pattern and therefore may vary with task requirements. For example, when investigated under different lighting conditions, visual cortex excitability, as indicated by the perception of phosphenes, is optimally enhanced at beta frequencies (14–22 Hz), whereas alpha frequencies (8–14 Hz) induce the most effective stimulation when applied in darkness (Kanai et al., 2008). Despite these results, the cognitive effects of TACS have received minimal attention and thus remain poorly understood. The different types of TES allow the administrator to flexibly choose between applying excitatory and inhibitory stimulation with TDCS, inducing random noise in TRNS, or altering brain oscillations with TACS, depending on the desired cognitive outcome.

## 3 Improving cognitive training using TES in the adult brain

The goal of cognitive training is to improve a targeted cognitive function, if possible to the optimal degree. The outcome may be different from individual to individual. However, performance needs to be quantifiable to measure the effect, especially in order to assess the effect of TES. Most training studies compare reaction time and/or accuracy pre- and post-training to monitor the success of the intervention. In addition, it is desirable that follow-up measures should be taken post-training to ascertain long-term effects and to allow flexible re-application of the training if necessary. Additional tasks using non-practiced material can be applied pre- and post-training to examine whether the trained material generalises to broader cognitive performance in the same or other domains.

Working memory, attention, language, and visual processing are frequently reported as cognitive training targets in TES studies (Table 3). Many of these studies have achieved significant improvements in performance compared to cognitive training alone under sham conditions. Some of this research is directly related to clinical conditions, such as language impairments (Table 2). For instance, in various TES studies participants were trained on the memorization of words (Flöel et al., 2008), repeated naming of objects presented in a picture (Holland et al., 2011), or reading practice (Turkeltaub et al., 2011). Even short training periods (e.g., 20 min) can significantly improve cognitive performance (e.g., Berryhill et al., 2010, Cattaneo et al., 2011, Flöel et al., 2008, Gladwin et al., 2012, Holland et al., 2011, Sandrini et al., 2012, Teo et al., 2011). Such an effect might be mediated by changes in the functional architecture of the brain, as those can already be observed after only a few minutes of TES (Chaieb et al., 2009, Polanía et al., 2012).

At the moment, it is still unclear what relevance the observed improvements will have in a real-life setting outside the testing laboratory. For example, the average improvement in reaction times for different types of cognitive processing is often below 70 ms compared to sham stimulation, which might be meaningless for most real-life situations (Pascual-Leone et al., 2012). However, many TES studies also report significant improvements in accuracy (e.g., Ferrucci et al., 2008, Fiori et al., 2011, Flöel et al., 2008, Marangolo et al., 2011, Ohn et al., 2008, Turkeltaub et al., 2011).

According to Pascual-Leone et al. (2012), scientific issues such as small and biased samples (i.e. often dominated by university students), behavioural detriments induced by the stimulation, and publication biases need to be addressed for a more qualitative evaluation of experimental outcomes (Iuculano and Cohen Kadosh, 2013). Weak or null effects may never make it into the pool of scientific literature, despite their importance for the critical evaluation of the method's effects. In the absence of effects, it is uncertain whether the method itself lacks the potential for cognitive enhancement, or whether the task and/or stimulation site may be irrelevant for the cognitive function in question. It is therefore necessary for researchers to also publish unsuccessful experiments and to carefully monitor previously used stimulation parameters and cognitive tasks in relation to the stimulated region in order to ascertain unbiased results. These parameters could be changed and adapted in future studies. For example, stimulating the IPS in a subject with low numerical abilities during numerical training may not optimize cognitive training because the subject might apply different strategies than other individuals of the same age; the subject may therefore recruit frontal regions, instead, for the same type of processing (Rivera et al., 2005). Similarly, it is possible that changes in other parameters such as the current intensity (e.g., from 1 mA to 1.5 mA) will lead to stronger effects (Teo et al., 2011).

Another important consideration for TES application and research is that the effect of ATDCS versus CTDCS can result in opposing directions of behavioural effects in different brain regions and for different cognitive tasks (Table 2, Table 3). For instance, CTDCS has been found to decrease memory performance when applied to temporo-parietal regions (Ferrucci et al., 2008), and similarly working memory recognition when the inferior posterior parietal cortex was stimulated (Berryhill et al., 2010). On the contrary, CTDCS to the right posterior parietal cortex has improved measures of attention (Weiss and Lavidor, 2012; for examples in other domains see Antal et al., 2004, Dockery et al., 2009, Terhune et al., 2011). The difference may be explained by the nature of the cognitive function subserved by the stimulation region, such that areas in which higher activation correlates with superior performance show performance enhancement when ATDCS is applied, whereas areas involved in attentional regulation might benefit from inhibition. In the latter case CTDCS would act to induce an attentional filter (Weiss and Lavidor, 2012).

In summary, the interpretation of results in TES research is often complex and may be counterintuitive in certain cases. This is mainly due to a lack of understanding of the exact interaction of effects between the cognitive function and the region to be stimulated. Such ambiguities might be partly resolved by systematically varying stimulation parameters and adding a wider range of testing materials to study designs using TES. So far, current findings in adults indeed indicate that various cognitive training effects can be significantly enhanced with TES compared to cognitive training alone. TES combined with cognitive training showed positive results with moderate to high effect sizes, even within stimulation periods as brief as a single session (Table 2, Table 3; see also Jacobson et al., 2012b). This provides support for the potential efficacy of this technique. However, the true nature of the cognitive outcome in real-life situations, especially in cognitive deficits, still needs to be established. Study designs need to be improved in the future in order to guarantee unbiased and satisfactory outcomes leading to clear interpretations for such studies. Current interpretations of findings therefore look promising but need to be critically evaluated.

## 4 Targeting learning difficulties in the developing brain

Based on the results from TES in the adult brain, we suggest that by targeting brain regions that subserve the impaired cognitive skills during cognitive training, the atypical trajectory of neural development in learning difficulties may be altered in the short term. In the long term, TES may thereby be effective for ameliorating the brain's plasticity constraints on learning, and potentially restoring normal learning processes and a more typical developmental pathway. We hereby provide a new perspective for developmental cognitive neuroscientists, and suggest that TES can have the potential to address both the neural and behavioural level of child learning difficulties.

Even though there is substantial overlap in the symptom profiles of different learning difficulties (Gilger and Kaplan, 2001), it has also been suggested that learning difficulties often exhibit more specialized impairments (Scanlon, 2013). As an example, we will focus here on DD, which involves problems in relating magnitudes to spatial representations along a mental number line (e.g., 20 is larger than 18 but smaller than 25) (Ashkenazi and Henik, 2010, Von Aster and Shalev, 2007). This lack of understanding of numbers might contribute to broader issues with mathematical thinking and should be targeted by training that attempts to alleviate effects of DD. Indeed, number line training has been shown to improve spatial representations and improved arithmetic performance in children with DD (Kucian et al., 2011). A recent study in young adults provided proof of concept that TES during artificial number training improved performance on the number line task after 6 days of training (Cohen Kadosh et al., 2010). The TES effect remained during a follow-up test 6 months after the end of the training. The persistence of the TES training effect suggests the possibility of TES improving cognitive functioning with long-term effects. Notably, recent studies in the field of numerical cognition have shown similar long-term effects after arithmetic training (Snowball et al., 2013), fraction training (Looi et al., 2013), as well as numerosity discrimination training (Cappelletti et al., 2013).

It is important to mention that we do not intend in any way to belittle the effect of cognitive training itself. Cognitive training alone has been shown to induce a certain amount of change at both performance and neural levels (e.g., Krafnick et al., 2011, Kucian et al., 2011, Takeuchi et al., 2011). However, current results indicate that electrical stimulation can enhance training effects with measurable changes in the brain by modulating neurochemicals that are involved in LTP (Fritsch et al., 2010, Stagg et al., 2009) and may therefore prime it for neuroplasticity. This may subsequently optimize the effect of the training. This could optimize the cognitive benefits of training, especially for skills subserved by dysfunctional networks, which otherwise are too weak to unfold the full learning potential. Since in these cases the brain is suboptimally developed at anatomical and/or functional levels (Gilger and Kaplan, 2001), TES could facilitate synaptic strengthening in the stimulated neural circuit during and after training, by the mechanisms described earlier (Section 2.1). It has been suggested that by facilitating synaptic strengthening, the child's cognitive potential could be increased (Holt and Mikati, 2011) and may even exceed the limits that were initially imposed by the learning difficulties.

Learning difficulties are associated with complex patterns of brain atypicality at both functional and structural levels (e.g., DD: Kucian et al., 2006, Mussolin et al., 2009, Price et al., 2007, Rykhlevskaia et al., 2009; dyslexia: Stein and Walsh, 1997, Temple et al., 2003; ADHD: Shaw et al., 2012). Successful cognitive training itself, when applied during sensitive periods of plasticity, has the potential to alter or even redirect atypical brain functioning and thereby possibly promote structural reorganization (Knudsen, 2004). Unfortunately, extensive training cannot always be provided at the right time. Due to factors such as late identification, training might occur after the end of a sensitive period, yet one would still want to maximize the success of cognitive training. Moreover, conventional cognitive training programmes have several limitations. First, school-based intervention programmes are costly and time-consuming (e.g., the annual cost for one-to-one tutoring in numeracy or literacy is ∼£2500 per child in the UK (Gross et al., 2009)). Second, typical training intervention spans several months and adds an additional workload to the child's existing schoolwork, which may increase emotional and cognitive strains on the child. Third, a lengthy intervention programme also adds to the workload of staff involved in the intervention, and may limit the amount of children that receive intervention. The combination of both financial and training resources is not always available for each child, and therefore limits the possibilities for receiving lengthy cognitive training (Rabipour and Raz, 2012).

These caveats could be circumvented by introducing new ways to directly affect neuroplasticity in the deficient neural networks, in order to enhance the effect of cognitive training and lead to similar outcomes in a shorter intervention period. Similar motivation has driven the usage of TES with neurological patients (Holland and Crinion, 2012). TES seems sufficiently potent to induce such plastic neural changes and may thereby be likely to improve cognition in the long-term, as it frequently modulates neuronal excitability in a task-dependent way (Cohen Kadosh, 2013, Miniussi et al., 2008). In addition, learning difficulties are suitable for TES treatment, as their underlying brain atypicalities involve mainly cortical regions that are accessible to TES (Wagner et al., 2007).

Recent studies on adults have found that TES modulates the stimulated brain region (Holland et al., 2011), as well as the network the stimulated region is part of (Keeser et al., 2011, Zheng et al., 2011). Therefore, while TES modulates the cortical excitability of the stimulated brain region, the simultaneously administered TES and cognitive training may lead to the strengthening of weak connections within the deficient network by lowering the neuronal threshold and repeatedly activating the network. Since the integration and specialization of neural circuits throughout development involve the interaction of different brain areas, including their excitatory and inhibitory interconnections (Cohen Kadosh, 2011, Johnson, 2011), the potential to modulate the functioning of local, as well as global network functioning, is very appealing. This means that TES aims to modulate the functioning of a deficient cortical region but affects the entire circuit involved in the processing of the task. The selective choice of the cognitive training in combination with the choice of the cortical region to be stimulated may determine which cognitive function and which neural circuit will be affected.

For instance, research in healthy adults and clinical populations has shown that cognitive training paired with brain-targeted intervention can maximize neuroplasticity and thereby significantly improve behavioural performance (for reviews see Cohen Kadosh, 2013, Holland and Crinion, 2012, Jacobson et al., 2012b), including long-lasting effects (Cohen Kadosh et al., 2010, Reis et al., 2009). Longer-lasting effects yet need to be established. Especially for paediatric populations, long-term follow-ups over several years of development will be both necessary and valuable to gain an understanding of long-term TES effects on the developing brain.

To summarize, the neuroplasticity induced by TES could support and complement the cognitive training effects and potentially even adapt or redirect the ill-defined developmental trajectories of underdeveloped brain regions. The burden on the individual child, the child's immediate and social environment, and the economic burden could all be mitigated in this way. The potential for enhancing plasticity may also benefit different age groups (e.g., adults) and different profiles of cognitive impairments. Currently, this is a future perspective and further research needs to establish the grounds for such an application in order to make it safe and effective. Despite the promising potential of TES to improve cognitive functions, it is also important to consider its potential risks and pitfalls in stimulating the child brain.

## 5 Potential risks and pitfalls of TES

Even though there is increasing evidence for the efficacy of TES in improving cognitive functions, it is important to consider its possible physical and psychological side effects, especially when applied to children.

## 6 Physical side effects

Seizure induction is currently considered the most critical and hazardous possible consequence resulting from brain stimulation. Researchers must be aware that side effects in children might be either qualitatively or quantitatively different from those observed in adults, and that critical evaluation of pre-existing health conditions in children that might impact the effect of TES is essential. It will be necessary to ascertain and possibly extend screening measures and current exclusion criteria used for adults to exclude participants with a family history of epilepsy or other neurological and psychiatric disorders. Even then, a residual risk of the child unknowingly being prone to seizure activity will remain. It is also highly important that parents and children undergoing TES are well-informed and understand the potential risks and what these might pose to the child's health.

Since the electrical current of TES is kept very low (e.g., 1–2 mA), the risk of major physical side effects such as tissue damage is considered unlikely and has not yet been observed to occur in experimental settings (Brunoni et al., 2012). Some of the few reported minor physical side effects during stimulation involve tingling, itching or a burning sensation of the skin under the electrode and in rare cases discomfort, including slight nausea and headaches (Poreisz et al., 2007; Table 1). Most studies, including modelling work such as current density magnitude evaluation, have thus far included only adults. Since the child central nervous system might respond differently to TES (e.g., due to smaller distances between the scalp and the brain tissue, or due to differences in the organization of gyri and sulci), the potential physical side effects of TES cannot be entirely anticipated at this point. Currently, there have been only a limited number of published TES studies involving atypically developing children and adolescents (Mattai et al., 2011, Schneider and Hopp, 2011), which have mostly confirmed their tolerability for TES. Neither study reported any significant physical side effects during or after TES treatment.

The current gap in paediatric research can most likely be explained by the lack of experience and consequential systematic avoidance of non-invasive brain stimulation techniques in children for two reasons: (1) researchers in developmental cognitive neuroscience might be unaware of TES and of the current findings of improving cognitive learning and training in adults using TES; (2) concerns of causing irreversible changes in the developing brain might deter scientists, as the current lack of paediatric research in this domain inflicts a significant amount of responsibility on the researcher.

## 7 Cognitive side effects

While cognitive side effects of TES have hardly been examined at this point, clinicians and researchers should be aware of potential risks and the relative lack of systematic research in this field when applying the method.

Firstly, the developing brain represents a ‘flexible target’ and due to continuous plastic changes in both brain structure and function, the ideal brain regions for stimulation in the individual learning difficulties are unknown and might change with development. Typically developing children recruit different brain regions or use the same regions but to a different extent and at different time points in development, and apply different cognitive strategies, than adults (Cohen Kadosh et al., 2012a, Cohen Kadosh, 2011, Jolles and Crone, 2012). During normal development, for instance, arithmetic abilities shift from recruiting mainly frontal regions at younger ages to relying more on parietal regions later in life (Rivera et al., 2005). The exact time of the shift can hardly be predicted in typically developing children and might be even less predictable in children with atypical development. In addition, the atypically developing brain might respond and adapt differently to the stimulation. This complex issue requires especially careful scientific exploration and attention. Experimental data is required to assess what time during development would benefit the cognitive deficit most and whether certain periods during development should even be avoided. In addition, more knowledge on the exact developmental trajectory in learning difficulties is needed, but the current consensus of data can serve as a starting point for TES study designs. In this respect, the synergy between cognitive training, neuroimaging and TES studies will help acquire knowledge not only about the neural correlates of cognitive development, but also about the direct causal relationship between the function of a given brain area (using TES) and the acquisition of cognitive abilities. This in turn will have practical implications for both applied and basic sciences on intervention in atypical brain development.

Secondly, remote regions may be modulated by the relocation of blood flow and energy supply to the stimulated brain area. For instance, haemodynamic changes after TDCS were found to be altered in the targeted region (Holland et al., 2011), but also in more distant brain regions that were functionally related to the stimulated region (Keeser et al., 2011, Zheng et al., 2011). The stimulation of a particular brain area (e.g., Broca's area) along with the associated cognitive enhancement (e.g., language) might thereby reduce the cognitive functions of other domains that are subserved by proximal brain regions (e.g., cognitive control in the dorsolateral prefrontal cortex (Brass et al., 2005)). A recent study has shown that this scenario is possible. In this study, TES to the posterior parietal cortices improved artificial number learning but impaired automaticity on the learning task, whereas TES to the dorsolateral prefrontal cortices impaired the learning but improved automaticity of artificial number learning (Iuculano and Cohen Kadosh, 2013). Especially during brain development, the balance between different brain areas might be more easily disturbed by changes induced by TES and training.

Effects of TES on the untrained cognitive functions need to be examined in addition to the specific target function, to ascertain that enhancing one cognitive domain will not impair another. Therefore, it is important to assess a larger range of functions (e.g., attention, working memory, executive functions, social skills) rather than merely the domain of interest. This is not only of interest immediately after the training but also for follow-up tests that assess long-term cognitive effects. It is therefore highly important for the scientific community to critically assess and monitor long-term effects of TES.

We would like to stress that we do not regard TES as a potential panacea that can solve all possible cortical deficiencies by enhancing neuroplasticity in general. Instead we argue that it is important to examine whether TES can be a successful support for cognitive training in children with atypical cortical functioning. This in turn, will add value to current interventions, even potentially for cognitive functions and deficits that have not yet been considered with this method. Even if it proves successful in one form of atypical brain development, it might show either negative, positive or no effects in other domains. The complex interactions of affected brain circuits are too diverse across disabilities to generalize result interpretations from one to the other. The success of TES in each individual developmental disability or disorder is thereby by no means guaranteed but needs to be empirically established.

While we acknowledge the potential risks of TES, neglecting it as a possible method to improve the cognitive learning and subsequently the lives of large numbers of children and adults, might be considered as an ethical failure (Cohen Kadosh et al., 2012b). We suggest that the first stage of research should involve small sample sizes of children with learning difficulties that should be monitored for behavioural changes and improvements post-treatment, before conducting studies that will involve larger samples. This in turn will assist in assessing the potential of TES as an intervention method in children with learning difficulties in clinical settings.

In order to protect paediatric participants from any potential harm that could be caused by TES in research, minimal risk standards need to be established by review boards. The risk exposure should be based on weighing possible negative consequences against the benefit for the individual child (Wendler and Varma, 2006).

## 8 Guidelines

Cohen Kadosh and colleagues discuss the subject of ethics in child brain stimulation in more detail (Cohen Kadosh et al., 2012b) and offer several potential solutions to issues arising from bringing TES into clinical settings. For instance, the use of TES machines needs to be restricted to prevent premature use by unqualified or inadequately trained individuals, including parents. Overly concerned or motivated parents might be tempted to purchase a stimulator, which is nowadays publicly available online, in order to train their children at home without the required knowledge about the necessary cognitive training, or the parameters and sites for stimulation and thereby cause no effects, or even physical and cognitive impairments. In order to avoid such premature use, we suggest that training needs to be required for practitioners, in order to restrict the use of TES to professionals. This is not yet the case, such that the current use of TES does not follow any formal regulations. If TES gains popularity in the general society, it might also be advisable to publicly warn and educate people of the danger of using TES without the required technical knowledge and awareness of its risks.

Moreover, one of the misconceptions about TES is that it improves cognitive performance by itself. In fact, the opposite is the case: it is essential to combine TES with the appropriate cognitive training, and to apply it to the correct brain region, in order to use its full potential to improve cognitive performance (Reis and Fritsch, 2011). Careful education and communication with the families and the child's general practitioner prior to the treatment is essential for research experiments. It is important that children eligible for TES studies will understand what the procedure they are undergoing entails, and what they give their assent to.

## 9 Conclusion

Research from various laboratories worldwide, using a range of parameters and involving different cognitive domains, has shown that in many cases, TES coupled with cognitive training can improve cognitive training effects in both healthy and clinical adult populations. This suggests that TES may be a useful aid in promoting cognitive training effects where there are atypicalities in the brain that are otherwise not optimally addressed by cognitive training alone. However, the step from this adult research to child populations and atypical development still needs to be made. We suggest that with several precautions taken, TES can serve as a successful addition to cognitive training for learning difficulties. Such precautions include the careful investigation of both short- and long-term effects of TES on the atypically developing brain, as well as the introduction of guidelines and restrictions for the use of TES in paediatrics for researchers and licensed professionals only.

Current findings need to be viewed from a critical perspective and research designs in the future have to be adjusted and refined in order to provide consistent and satisfactory outcomes with relevance for real-life outcomes. Closing the current gap in developmental cognitive neuroscience in respect to TES, along with its potential effects and possible risks, will allow us to assess whether optimizing cognitive training of individuals with atypical brain development using TES is achievable. This will thereby help to devise new ways to reduce the severe consequences of cognitive disability on the individuals’ lives, their families, and society.

## Conflict of interest statement

R.C.K. is a Wellcome Trust Career Development Fellow (0883781). R.C.K. filed a patent for an apparatus for improving and/or maintaining numerical ability.
